# Calcium-binding protein S100P is a new target gene of MACC1, drives colorectal cancer metastasis and serves as a prognostic biomarker

**DOI:** 10.1038/s41416-022-01833-3

**Published:** 2022-05-21

**Authors:** Felicitas Schmid, Mathias Dahlmann, Hanna Röhrich, Dennis Kobelt, Jens Hoffmann, Susen Burock, Wolfgang Walther, Ulrike Stein

**Affiliations:** 1 Charité – Universitätsmedizin Berlin, corporate member of Freie Universität Berlin and Humboldt-Universität zu Berlin, Berlin, Germany; 2 Experimental and Clinical Research Center of the Charité – Universitätsmedizin Berlin and the Max-Delbrück-Center for Molecular Medicine in the Helmholtz Association, Robert-Rössle-Straße 10, 13125 Berlin, Germany; 3 German Cancer Consortium (DKTK), Im Neuenheimer Feld 280, 69120 Heidelberg, Germany; 4 Experimental Pharmacology and Oncology Berlin-Buch GmbH, Robert-Rössle-Straße 10, 13125 Berlin, Germany; 5 Charité Comprehensive Cancer Center, Invalidenstraße 80, 10117 Berlin, Germany

**Keywords:** Colon cancer, Tumour biomarkers

## Abstract

**Background:**

The metastasis inducing gene MACC1 is a prognostic and predictive biomarker for metastasis in several cancers. Its mechanism of inducing metastasis includes the transcriptional control of other cancer-related target genes. Here, we investigate the interplay with the metastasis driver S100P in CRC progression.

**Methods:**

MACC1-dependent S100P expression was analysed by qRT-PCR. The binding of MACC1 to the S100P promoter was determined by ChIP. Alterations in cell proliferation and motility were determined by functional in vitro assays. In vivo metastasis after intrasplenic transplantation was assessed by bioluminescence imaging and evaluation of tumour growth and liver metastasis. The prognostic value of S100P was determined in CRC patients by ROC-based Kaplan–Meier analyses.

**Results:**

Expression of S100P and MACC1 correlated positively in CRC cells and colorectal tumours. MACC1 was found binding to the S100P promoter and induces its expression. The overexpression of S100P increased proliferation, migration and invasion in vitro and significantly induced liver metastasis in vivo. S100P expression was significantly elevated in metachronously metastasising CRC and was associated with shorter metastasis-free survival.

**Conclusions:**

We identified S100P as a transcriptional target gene of MACC1. Expression of S100P increases the metastatic potential of CRC cells in vitro and in vivo, and serves as a prognostic biomarker for metastasis-free survival of CRC patients, emphasising novel therapeutic interventions targeting S100P.

## Background

Colorectal cancer (CRC) is one of the most common cancers and is the second leading cause of cancer-related deaths [1]. The formation of distant metastases severely reduces the survival of CRC patients [2], defining a clinical need for prognostic and causal biomarkers to evaluate the risk of metastasis formation and novel therapeutic intervention points.

Metastasis associated in colon cancer 1 (MACC1) was identified as a causal driver for metastasis formation in CRC. High expression levels of MACC1 promote cell proliferation, motility and drug resistance, thereby facilitating cancer progression and metastasis [3]. It is confirmed as a prognostic and predictive biomarker in a variety of solid cancers like CRC, gastric, oesophageal, pancreatic, hepatocellular/biliary, lung, ovarian, breast, renal, bladder, nasopharyngeal cancer, glioblastoma and osteosarcoma [3, 4]. MACC1 is directly associated with the regulation of c-MET expression, subsequently inducing EMT via elevated HGF/c-MET signalling [5, 6], but also of the pluripotency marker NANOG [7] and the cell adhesion factor SPON2 [8], both contributing to cancer progression and metastasis.

Among other metastasis drivers, S100 calcium-binding protein P (S100P) has been reported to increase cell proliferation and motility, and thus the progression of several solid cancers [9, 10]. Overexpression of S100P has been found to promote metastasis of a benign rat mammary cell line [11], and further studies link S100P mechanistically to increased migration of individual cells, as well as collective cell invasion [12, 13].

Further, S100P serves as a prognostic biomarker for gastrointestinal cancers, including CRC [14, 15].

Both biomarkers, MACC1 and S100P, play a role in CRC progression and liver metastasis (summarised by Weidle et al. [16]), but a mechanistic connection of both factors has not been described so far. In this study, we identify S100P as a transcriptional target gene of MACC1. We show that the expression of S100P increases cell proliferation and motility in vitro and drives CRC metastasis in vivo, and we assess its value as a prognostic biomarker for metachronous metastasis of CRC patients.

## Methods

### Plasmid generation

The full-length cDNA of S100P (Horizon Discovery, Cambridge, UK) was cloned into the mammalian expression vector pcDNA3.1 (Invitrogen, Carlsbad, MA), introducing a C-terminal V5-6xHis tag. Empty pcDNA3.1 vector was used as a negative control. For the RNAi-based knockdown of S100P, expression oligonucleotides for targeting S100P mRNA sequence (5’-aatggagatgcccaggtggactctcttgaagtccacctgggcatctcca-3’) were hybridised and cloned into the pSil4.1 expression vector using the pSilencer neo Kit (Invitrogen). A plasmid containing unspecific control shRNA was used as a negative control.

### Generation of modified cell lines

Human CRC cells, derived from the cell lines SW480, SW620 and HT29, were grown in RPMI-1640 or DMEM medium (PAA Laboratories, Pasching, Austria), containing 10% foetal bovine serum (FBS, PAA Laboratories), respectively, and were cultured in a humidified atmosphere with 5% CO_2_. Authentication of cells was performed by short tandem-repeat (STR) genotyping (DSMZ, Braunschweig, Germany). STR genotype was consistent with published genotypes. The generation of SW480/MACC1 and SW480/vector cells was previously described [17]. The cell lines SW620/shMACC1 and SW620/shCtrl were generated by transfection of the respective gene-specific shRNA expression constructs as previously described [18]. HT29 cells with stable expression of GFP (HT29/GFP) and MACC1-GFP (HT29/MACC1-GFP) were kindly provided by Benedikt Kortüm (Max-Delbrück-Center for Molecular Medicine, Berlin, Germany). Transfection of SW480 cells with the pcDNA3.1 vector alone or with inserted S100P cDNA resulted in the cell lines SW480/vector and SW480/S100P, respectively. Both cell lines were later transfected with a luciferase-expressing plasmid [18], generating SW480/luc/vector and SW480/luc/S100P cells. Equal luciferase activities were verified using the Steady-Glo Luciferase Assay System (Promega, Madison, WI). SW620/shS100P and SW620/shCtrl cells were generated by transfection of pSilencer vectors harbouring S100P-specific or unrelated control shRNA sequences, respectively. Stable clones of transfected cell lines were selected using 1 mg/ml neomycin (G418, PAA Laboratories) or 1 μg/ml puromycin (Clontech Laboratories, Mountain View, CA), respectively, for 6 weeks. All cells were regularly tested for the absence of mycoplasma using the MycoAlert mycoplasma detection kit (Lonza, Basel, Switzerland). Detailed information on the generated cell lines is listed in the Supplement (Supplementary Table S1).

### RNA isolation and quantitative real-time reverse transcription-PCR (qRT-PCR)

Total RNA was extracted with the Universal RNA Purification Kit (Roboklon, Berlin, Germany) and reverse transcribed. In total, 20 μl reactions with 50 ng of RNA, 1× RT Buffer, 1 U RNase inhibitor, 10 mM MgCl_2_, random hexamers, 250 μM pooled dNTPs and 2.5 U MuLV reverse transcriptase (all products from Applied Biosystems, Foster City, CA) were prepared. The RT reaction was carried out in a T300 thermocycler (Biometra, Göttingen, Germany) for 15 min at 42 °C, with 5 min at 95 °C for deactivation of the enzyme and final cooling to 4 °C. qRT-PCR was performed in duplicate with the GoTaq qPCR Master Mix (Promega) in a final volume of 10 μl in a LightCycler 480 (Roche, Basel, Switzerland). PCR protocol included a pre-incubation step at 95 °C for 2 min, followed by 45 cycles of incubation at 95 °C for 7 s, annealing at 60 °C for 10 s and elongation at 72 °C for 5 s. In the melting curve, the temperature was increased from 65 °C to 95 °C (0.1 °C/s). Data were analysed with the LightCycler 480 Software release 1.5.0 SP3 (Roche). MACC1 or S100P expression was normalised to the expression of GAPDH. The following gene-specific primer were used: MACC1_fow 5’-ttcttttgattcctccggtga-3’; MACC1_rev 5’-actctgatgggcatgtgctg-3’; S100P_fow 5’-aatctagcaccatgacgg-3’; S100P_rev 5’-tctgcaggaagcctggta-3’; GAPDH_fow 5’-gaagatggtgatgggatttc-3’; GAPDH_rev 5’-gaaggtgaaggtcggagt-3’.

### Protein extraction, extracellular protein assay and western blotting (WB)

Protein extraction of trypsinised cells from regular cell culture occurred by 30-min lysis in RIPA buffer on ice. The protein content of each sample was determined and equal amounts were denatured for 5 min at 95 °C in the presence of 1× NuPAGE sample buffer (Invitrogen), supplemented with 10% DTT. Proteins were separated by SDS-PAGE and transferred onto a Hybond C Extra nitrocellulose membrane (Amersham Biosciences, Little Chalfont, UK). The membrane was blocked with 5% non-fat dry milk in Tris-buffered saline with Tween 20 (TBST) and incubated overnight with anti-human MACC1 (1:1000 dilution; Sigma-Aldrich) or S100P (1:250 dilution; R&D Systems, Minneapolis, MN), with β-actin (1:20,000 dilution; Pierce) as a loading control. The membrane was washed, incubated for 1 h with an anti-rabbit HRP-conjugated (1:10,000 dilution; Promega) antibody. Finally, the membrane was washed again and incubated with ECL solution (WesternBright ECL kit; Advansta, Menlo Park, CA). Protein bands were detected by using CL-XPosure films (Thermo Fisher) with exposure times between 1 s and 20 min. Experiments were carried out three independent times.

### Enzyme-linked immunosorbent assay (ELISA)

To analyse secreted S100P protein, of each analysed cell line, 2 × 10^6^ cells were seeded in six-well plates and grown for 24 h in the respective medium containing 10% FCS. Cells were washed and incubated with serum-free medium (2 ml) for another 24 h, before the medium was removed and gently spun down to remove detached cells. Quantitative analysis of secreted S100P protein was performed with protein-specific ELISA (Human S100P ELISA; RayBiotech), according to the manufacturer’s instructions. Data acquisition occurred in duplicates in three independent experiments. The calibration curve of the ELISA and the quantified amounts of extracellular S100P in the growth media are listed in the supplement (Supplementary Tables S2 and S3).

### Chromatin immunoprecipitation (ChIP)

ChIP was carried out using the EZ-ChIP Kit (Millipore, Burlington, MA) according to the manufacturers’ instructions. SW480/vector and SW480/MACC1 cells were cross-linked, harvested, lysed and incubated with an anti-V5-tag antibody (Abcam, Cambridge, UK) against the overexpressed MACC1- V5-tagged protein or unspecific human IgG. The promoter regions of S100P or GAPDH were amplified by PCR using the PWO-Master (Roche) according to the manufacturers’ protocol and the following primers: S100Pp_fow 5’-ccgagacacaggtgaacac-3’; S100Pp_rev 5’-gtcatggtgctagattcagac-3’. GAPDH was used as an unrelated gene to validate the specificity of the observed binding. PCR products were separated by gel electrophoresis, and gel pictures were taken. ChIP assay was performed two independent times.

### Functional assays for cell proliferation and motility

For proliferation assays, 5 × 10^3^ cells were seeded into a 96-well plate. 3-(4,5-Dimethylthiazol-2-yl)-2,5-diphenyltetrazolium bromide (MTT, Sigma-Aldrich) was added with a final concentration of 0.5 mg/ml and cells were incubated for 2.5 h at 37 °C. Medium was removed and crystallised formazan salt was dissolved in DMSO. The plate was measured at 560 nm with an infinite M200 Pro plate reader (Tecan, Männedorf, Switzerland). Cell proliferation was repeated three independent times. Anchorage-independent growth was assayed as colony formation in soft agar plates. The lower layer contained 5% w/v agarose in RPMI-1640 medium with 10% FBS and the upper layer contained 4 × 10^4^ single cells in 0.33% w/v agarose and RPMI-1640 medium with 10% FBS. Cell colonies with more than four cells were counted by using a Leica DM IL microscope (×40 magnification; Leica Microsystems, Wetzlar, Germany). The experiment was done in triplicates and assays were carried out three independent times. Cell migration was assayed using transwell chambers with 12-μm pores (Corning, Corning, NY). In total, 2.5 × 10^5^ cells in 400 μl medium were seeded in triplicate into the upper chamber. The lower chamber was filled with 600 μl medium. Migrated cells were counted using Neubauer chambers after 24 h. Each experiment was performed three independent times. For wound-healing assays 4 × 10^5^ cells were seeded into a six-well plate. The cells were allowed to adhere for 24 h. The cell monolayer was wounded using small pipette tips. The wound was documented until day four and images were taken with a Leica DM IL microscope (×10 magnification). The assay was performed two independent times. For invasion assays, transwell chambers with 12-μm pores were coated with 60 μl of 1:3 diluted matrigel (BD Biosciences, San Jose, CA). In total, 5 × 10^5^ cells in 300 μl medium were seeded in triplicate into the upper chamber. The lower chamber was filled with 700 μl medium. After 72 h, cells in the lower chamber were harvested and counted with Neubauer chambers. Invasion assays were repeated three independent times.

### In vivo metastasis formation

The effect size of metastasis formation in intrasplenic xenograft mouse models was calculated with G'Power (v3.1.9.7; University Düsseldorf, Germany) and a sample size of nine animals per group was determined for a power of 80% for the animal experiment in this study. In all, 3 × 10^6^ SW480/luc/vector or SW480/luc/S100P cells (*N* = 9 mice, each) in 50 µl phosphate-buffered saline (PBS) were intrasplenically transplanted into severe combined immune deficient (SCID) beige mice (Charles River, Wilmington, MA). Bioluminescence measurements were performed once a week for a time period of 8 weeks. Mice received intraperitoneally 150 mg/kg luciferin (Promega), were anaesthetised with isoflurane (TH. Geyer, Berlin, Germany) and bioluminescence measurements were performed by using a Night Owl LB 981 imager (Berthold Technologies, Bad Wildbad, Germany) with an exposure time of 5 min. Mice Were sacrificed when the spleen tumour was palpable (on day 49) and spleens and livers were removed, documented, and liver metastases with a diameter larger than 1 mm were counted.

### CRC tissue samples, patients’ characteristics

The analysed CRC patient cohort (*N* = 60) without synchronous metastasis (UICC I–III) was previously described [6]. In brief, CRC patients were previously untreated, did not have a history of familial colon cancer, did not suffer from a second tumour of the same or a different entity and underwent surgical R0 resection. Consecutive tissue sections from formalin-fixed paraffin-embedded (FFPE) tumours were subjected to immunohistochemistry against MACC1 and S100P. Isolated mRNAs from microdissected tumour tissue samples were used for gene expression quantification via qRT-PCR, normalised to a calibrator. Follow-up survival data of the patients were used for ROC-based analysis of metastasis-free survival.

### Immunohistochemistry and imaging

FFPE tissue sections were xylene-fixed, de-paraffinated and blocked with PBS containing 2% horse serum. Tissue sections were incubated with MACC1 (1:500) and S100P (1:250) antibodies in TNT buffer (0.1 M Tris/HCl, pH 7.5; 150 mM NaCl; 0.1% Tween 20) overnight at 4 °C, washed in PBS and stained with the DAB/HRP kit (Vector Laboratories, Burlingame, CA), according to the manufacturer’s instructions. Nuclei were counterstained with haematoxylin/eosine. Pictures were taken with a BZ- X800 microscope (Keyence) at ×20 and ×40 magnification.

### Statistics

Statistical analysis was performed with PRISM v5 (GraphPad, San Diego, CA) or SPSS v18 (IBM, Armonk, NY). The comparison of two groups was done by a two-sided Student’s *t* test. The significance of box plots was tested by Mann–Whitney *U* test. The cut-off value of the S100P expression was determined by ROC (receiver operating characteristic) analysis. The Kaplan–Meier method was used to estimate cumulative survival rates, and differences in survival rates were assessed using the log-rank test. The odds ratios (OR) or hazard ratios (HR) with corresponding 95% confidence intervals (CI) were calculated. A *P* value below 0.05 was considered statistically significant.

## Results

### MACC1 regulates the expression of S100P in CRC cells

**Fig. 1:**
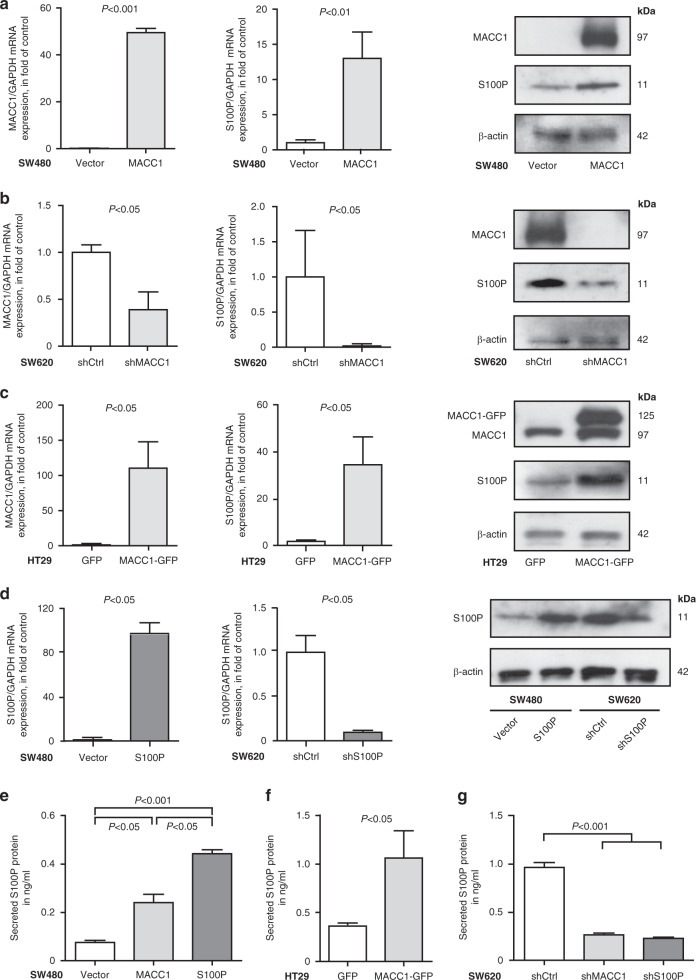
MACC1 regulates S100P expression in human CRC cells. **a**–**d** Expression levels of MACC1 and S100P were determined by qRT-PCR and WB. Data show average gene expression ± SEM; *N* = 3. GAPDH served as a house-keeping gene, β-actin as loading control. Ectopic overexpression of MACC1 (left) in SW480 cells (**a**) lead to an upregulation S100P mRNA (centre) and protein levels (right), compared to control cells. Gene-specific knockdown of MACC1 (left) in SW620 cells (**b**) also decreased the S100P expression on mRNA (centre) and protein level (right). Ectopic overexpression of MACC1-GFP (left) in HT29 cells (**c**) resulted in increased mRNA (centre) and protein levels of S100P (right). Ectopic expression (left panel) or RNAi-based knockdown (right panel) in SW480 and SW620 cells (**d**), respectively, resulted in differential S100P expression. **e**–**g** Secreted S100P levels in the media of the respective cell lines were quantified with target-specific ELISA (*N* = 4). Ectopic overexpression of MACC1, as well as S100P in SW480 cells (**e**) significantly increased the level of secreted S100P protein after 24-h incubation in serum-free medium, similarly to an ectopic expression of a MACC1-GFP fusion construct in HT29 cells (**f**). RNAi-mediated knockdown of MACC1, as well as S100P significantly reduced the amount of secreted S100P in the medium of the respective cells (**g**).

In a previous genome-wide expression analysis of an ectopic expression of MACC1 in the CRC cell line SW480, else virtually devoid of endogenous MACC1 expression, we found S100P highly upregulated (GSE70458; log_2_-fold change 6.23, adjusted *P* value <0.02) [8]. We validated the correlated expression of MACC1 and S100P on transcript and protein level, and observed a 13-fold upregulation of S100P expression in SW480/MACC1 cells, compared to control cells (*P* < 0.01; Fig. 1a).

While SW480 cells have been established from a primary colon adenocarcinoma, lymph node metastasis from the same tumour gave rise to the cell line SW620, which endogenously expresses high levels of MACC1, with higher capacity to migrate and metastasise. To validate the MACC1-dependent regulation of S100P expression in these cells, we also knocked down MACC1 in SW620 cells and observed a significant decrease of both MACC1 and S100P expression in SW620/shMACC1 cells (*P* < 0.05; Fig. 1b). When we analysed the overexpression of a MACC1-GFP fusion protein in addition in the unrelated CRC cell line HT29, we could validate the increase of S100P expression in HT29/MACC1-GFP cells, compared to HT29/GFP control cells (*P* < 0.05; Fig. 1c). To investigate the role of S100P on the metastatic potential of CRC cells, we generated stable cell lines with either ectopic S100P expression or shRNA-mediated knockdown of S100P in the isogenic cell lines SW480 and SW620, respectively (Fig. 1d). As both intracellular S100P expression levels and high abundance of extracellular S100P can promote tumour progression and metastasis [19], we tested if the MACC1-mediated expression of S100P in CRC cells also leads to an increase in extracellular S100P protein levels. Indeed, we observed a significant increase of S100P protein in growth media of SW480/MACC1 cells (0.25 ng/ml ±  0.03), compared to SW480/vector cells (0.07 ng/ml ± 0.01), but less than SW480/S100P cells with ectopic S100P expression (0.46 ng/ml ± 0.01; *P* > 0.001; Fig. 1e). Similarly, the ectopic expression of a MACC1-GFP construct in HT29/MACC1-GFP cells (1.07 ng/ml ± 0.28) significantly increased the S100P level in the medium, compared to the control cells (0.37 ng/ml ± 0.02); *P* > 0.05; Fig. 1f). In turn, RNAi-based knockdown of MACC1 (0.25 ng/ml ± 0.05) and S100P (0.21 ng/ml ± 0.06) in SW620 cells reduced the amount of secreted S100P protein in the medium of the respective cells significantly, compared to SW620/shCtrl cells (0.96 ng/ml ±  0.05; *P* > 0.001; Fig. 1g).

### Ectopic S100P expression promotes proliferation and motility of CRC cells

**Fig. 2:**
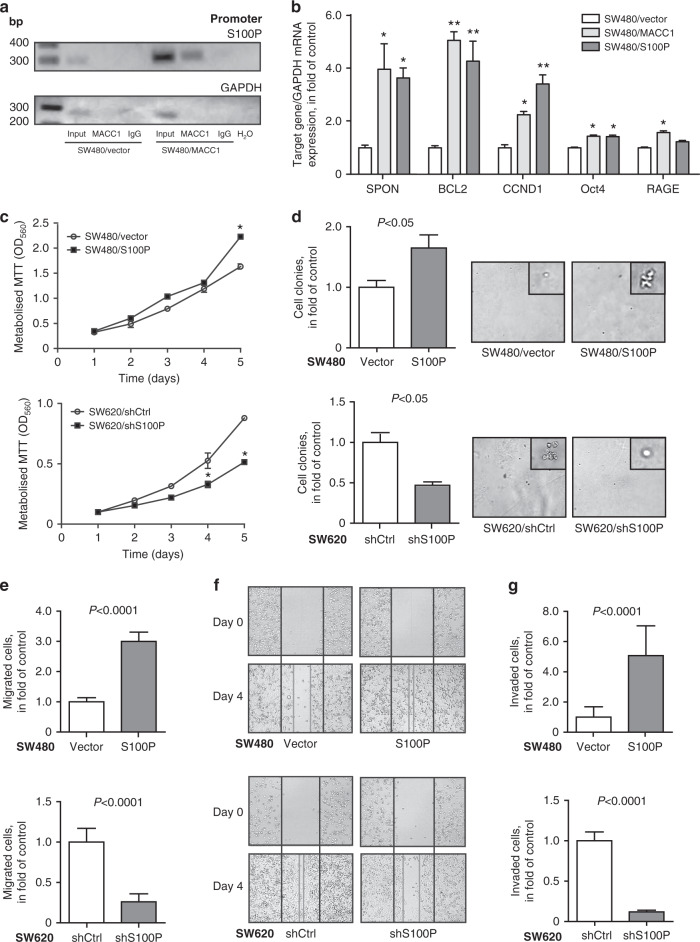
S100P promotes proliferation and motility of CRC cells. **a** Binding of MACC1 to the S100P promoter was determined by ChIP. A V5-specific antibody was Used to selectively pull-down ectopically overexpressed MACC1 in SW480/MACC1 cells (anti-V5). Pulling down V5 in SW480/vector cells or with unspecific human IgG in both cell lines (IgG) served as a negative control. Amplification of the S100P core promoter (−296 bp to +65 bp) indicates the binding of MACC1 to this fragment, while amplification of an unrelated GAPDH promoter sequence was used to test pull-down specificity. **b** MACC1- and S100P-related target genes were analysed in SW480/MACC1 and SW480/S100P cells by qRT-PCR and normalised to SW480/vector cells. Data show average values ± SEM; *N* = 3. **c** Elevated S100P expression increased the proliferation of SW480/S100P cells (upper panel), while knockdown of S100P decreased cell proliferation in SW620/shS100P cells (lower panel), compared to their respective control cells. Anchorage-dependent cell proliferation was determined by MTT assays. Data show average values ± SEM; *N* = 3. **d** Elevated S100P levels in SW480/S100P cells resulted in a higher ability of the cells to form colonies in soft agar (upper panel), while decreased S100P expression lead to fewer colonies of SW620/shS100P cells (lower panel), compared to their respective control cells. Single-cell suspensions were seeded in soft agar plates and colonies that were formed with more than four cells were counted. Representative images (×10 magnification) illustrate observed colony sizes. Data show average values ± SEM; *N* = 3. **e** S100P-overexpressing SW480/S100P cells showed higher cell migration through 12 μm pores of a transwell membrane, compared to SW480/vector cells (upper panel). SW620/shS100P cells with downregulated S100P expression migrated significantly less compared to control cells (lower panel). Cell migration was determined with Boyden chamber assays, counting the trans-migrated cells in the lower chamber. **f** SW480/S100P cells were able to close the wound within 4 days of incubation, compared to control cells (upper panel). The knockdown of S100P in SW620/shS100P cells resulted in decreased cell migration, in comparison to control cells (lower panel). In all, 300-µm wide wounds were inflicted in semi-confluent cell layers and wound closure was imaged daily. **g** SW480/S100P cells showed a higher ability to pass a matrigel-covered transwell membrane, compared to SW480/vector cells (upper panel). In turn, reduced S100P levels in SW620/shS100P resulted in decreased cell invasion, compared to control cells (lower panel). Data show average values ± SEM; *N* = 3.

In order to investigate the molecular mechanism of a MACC1-dependent regulation of S100P expression, we performed chromatin immunoprecipitation in SW480/MACC1 and SW480/vector cells, specifically precipitating ectopically expressed MACC1. Amplification of the S100P core-promoter region (−296 bp to +65 bp) [20] resulted in a strong PCR product after MACC1 precipitation in SW480/MACC1 cells, compared to its controls (Fig. 2a). We conclude that at higher expression levels, MACC1 is present in transcription complexes at the S100P promoter and contributes to its upregulation. Next, we were interested if there is an overlap of tumour-promoting target genes of MACC1 and S100P, when ectopically expressed in SW480 cells. Spondin 2 (SPON2) [8], the apoptosis regulator BCL2 [21], cyclin D1 (CCND1) [22] and the transcription factor Oct4 [7] were significantly overexpressed in both SW480/MACC1 and SW480/S100P cells, compared to SW480/vector cells (Fig. 2b), with a significant increase of CCND1 expression at overexpression of S100P compared to MACC1. SW480/MACC1 cells showed a significant decrease in Fas cell surface receptor (FAS) [21] expression, while the receptor of advanced glycation end products (RAGE) [23] expression was significantly increased in these cells (Fig. 2b). Subsequently, we evaluated the impact of elevated S100P expression on CRC cell growth and motility and observed an increase of anchorage-dependent cell proliferation in SW480/S100P cells (*P* < 0.05; Fig. 2c, upper panel), compared to SW480/vector cells. In turn, cell proliferation of SW620/shS100P cells with decreased S100P expression was significantly reduced (*P* < 0.05; Fig. 2c, lower panel), compared to SW620/shCtrl cells. Ectopic overexpression of S100P resulted in a 1.5-fold higher anchorage-independent colony formation of SW480/S100P cells, compared to SW480/vector cells (*P* < 0.05; Fig. 2d, upper panel). Similarly to the previously observed reduction in cell proliferation after knocking-down S100P, colony formation of SW620/shS100P cells was significantly decreased to 40% of the control cells (*P* < 0.05; Fig. 2d, lower panel). By determining alterations in cell migration after modulating S100P expression, we observed a threefold higher transwell migration of SW480/S100P cells, compared to SW480/vector cells (*P* < 0.0001; Fig. 2e, upper panel). In turn, cell migration of SW620/shS100P cells was reduced to 25% of SW620/shCtrl cells (*P* < 0.0001; Fig. 2e, lower panel). When assessing cell migration in wound-healing assays, SW480/S100P cells closed the inflicted wound 4 days, in contrast to SW480/vector cells (Fig. 2f, upper panel). In line with this observation, SW620/shS100P cells failed to close the wound within the same time (Fig. 2f, lower panel). Similarly to the previous migration assays, we performed cell invasion assays in Matrigel-coated transwells. SW480/S100P cells showed a fivefold higher cell invasion, compared to control cells (*P* <0.0001; Fig. 2g, upper panel). In turn, invasion of SW620/shS100P cells was decreased to 10% of SW620/shCtrl cells (*P* < 0.0001; Fig. 2g, lower panel).

### S100P overexpression drives metastasis formation in vivo

**Fig. 3:**
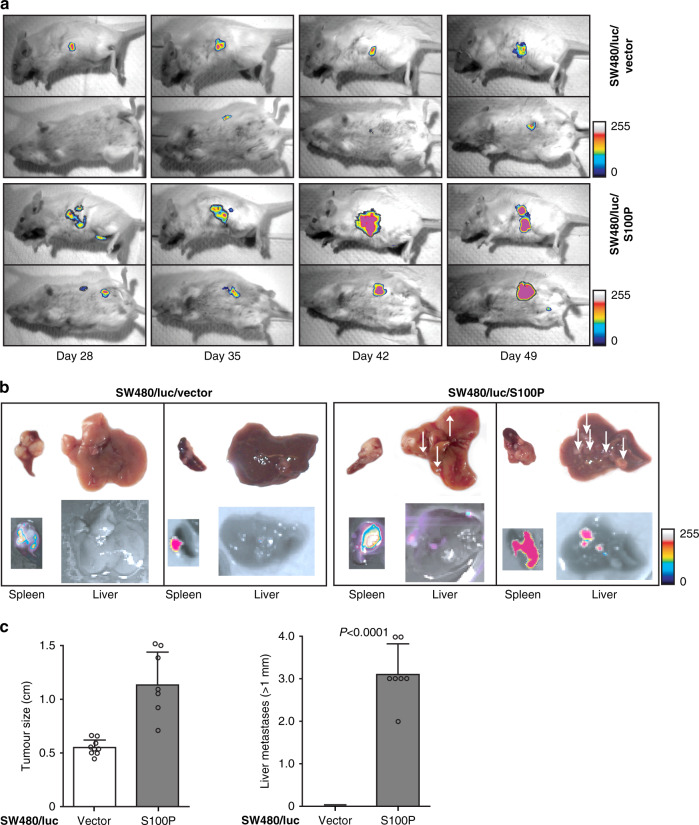
S100P overexpression drives metastasis formation in vivo. **a** SCID beige mice were intrasplenically transplanted with SW480/luc/vector upper panel) or SW480/luc/S100P cells (lower panel), with regular bioluminescence imaging to monitor tumour growth. **b** After sacrificing mice at day 49, spleens (tumour site) and livers (metastasis site) were removed and documented. Control mice (*N* = 7; left panel) did not develop liver metastases, while all mice with transplanted SW480/luc/S100P cells (*N* = 9; right panel) developed between one and six liver metastases. Ex vivo bioluminescence illustrated the tumour size and metastasis burden. **c** Quantification of tumour sizes showed an increased tumour growth (left panel), but also a significantly increased number of liver metastases (size <1 mm; right panel) for transplanted SW480/luc/S100P cells, compared to control cells. Data show average values ± SEM.

To evaluate the impact of S100P on tumour growth and metastasis formation in vivo by bioluminescence imaging, SW480/S100P cells and SW480/vector cells were stably transfected with firefly luciferase and subsequently transplanted into spleens of SCID beige mice. Mice were imaged once a week until the tumour burden reached an ethical limit (day 49). Tumour growth over time is exemplified by increasing bioluminescence signals of one sample per group (Fig. 3a). Spleens and livers of sacrificed mice were removed and analysed for tumour size and the number of distant metastases, respectively, exemplified by bright field and bioluminescence images (Fig. 3b). We observed moderately increased tumour growth in the group with transplanted SW480/luc/S100P cells, compared to transplanted SW480/luc/vector cells (Fig. 3c). But while no visible liver metastases were observed for the control group, an average of three liver metastases with a size above 1 mm in diameter were counted in animals with S100P-overexpressing tumours (*P*< 0.0001; Fig. 3c). The increase in cell proliferation and motility upon S100P expression in CRC cells, as shown above in vitro, did also enhance liver metastasis in vivo, validating the role of S100P as a metastatic driver.

### High expression of S100P correlates with metachronous CRC metastasis and decreased patient survival

**Fig. 4:**
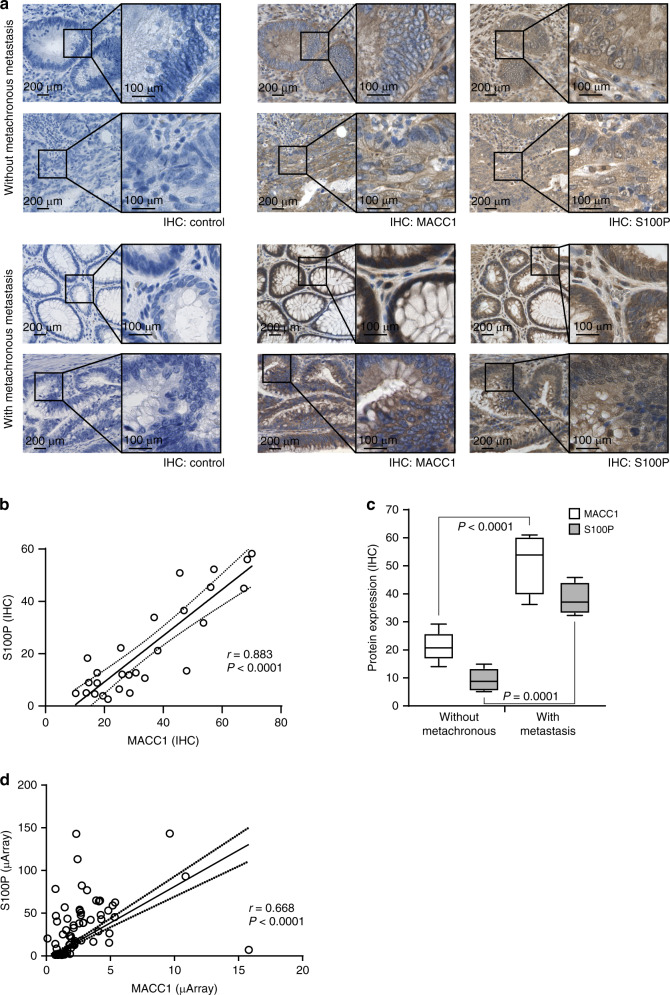
High S100P expression correlates with metachronous metastasis. **a** CRC tissue sections of primary CRC tumours without (upper panel) or with (lower panel) metachronous metastasis were analysed for MACC1 and S100P expression by IHC. Representative tumour regions illustrate the correlation of MACC1 and S100P expression at ×20 and ×40 magnification. **b** S100P protein expression correlates with MACC1 expression in the described CRC patient cohort after IHC staining (*N* = 27; solid line—linear regression; dotted line— 95% CI). **c** S100P protein expression is significantly increased in samples with high MACC1 expression. **d** S100P mRNA expression correlates with MACC1 expression in publicly available microarray datasets (*N* = 308; solid line—linear regression; dotted line—95% CI).

After observing increased metastasis formation of S100P-overexpressing cells in xenografted mice, we proved the potential use of S100P expression as a biomarker in human CRC patients. First, we stained MACC1 and S100P protein levels in tumour sections of CRC patients without synchronous metastasis at the time of diagnosis (UICC I–III) [6] by IHC (Fig. 4a). Sections from exemplary tumours without metachronous metastasis showed low MACC1, as well as low S100P IHC staining (Fig. 4a, upper panel). In contrast, we observed high MACC1 and elevated S100P IHC staining in tumour sections from CRC patients who suffered from metachronous metastasis (Fig. 4a, lower panel). Protein expression of MACC1 and S100P in the sections correlated significantly (*N* = 27; Fig. 4b) and we observed an increase of both MACC1 and S100P staining in tumour tissue with metachronous metastasis (Fig. 4c).

**Fig. 5:**
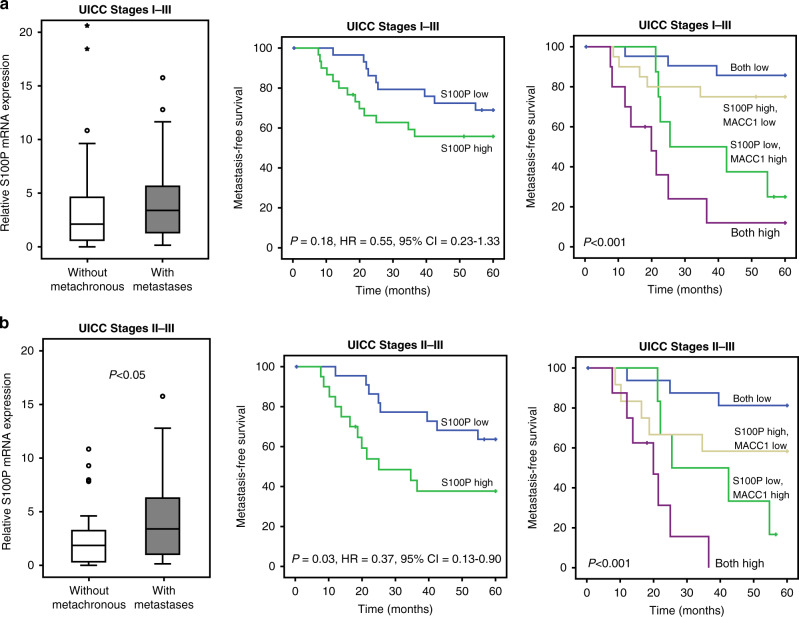
High S100P expression correlates with shorter metastasis-free survival in CRC patients. **a** S100P mRNA expression was found higher in tumours of patients (UICC I–III; *N* = 60) who developed metachronous metastases (left). Metastasis-free survival within 5 years after diagnosis was reduced when tumours express high S100P levels (centre). Combining MACC1 and S100P expression as prognostic biomarkers refines the identification of high-risk patients to develop metachronous metastasis (right). **b** Focusing on locally advanced tumours (UICC II–III; *N* = 43) the difference between tumours with and without metachronous metastasis became significant in S100P mRNA expression (*P* < 0.05; left), which corresponds to significantly lower metastasis-free survival (centre) of patients with high intratumoural S100P expression. The combination of MACC1 and S100P mRNA expression as prognostic biomarkers refines the identification of high-risk patients to develop metachronous metastasis (right).

The correlated expression of MACC1 and S100P was validated in publicly available microarray datasets of CRC tumours from the Gene Expression Omnibus repository. MACC1 and S100P transcript expression (*N* = 308) of MACC1 and S100P from the datasets GDS4393 [24], GDS4513 [25], as well as GDS4516 and GDS4718 [26] were merged after normalisation and also showed a significant correlation (Fig. 4d). In turn, we analysed the S100P expression in microdissected mRNA samples of CRC patients without synchronous metastasis at the time of diagnosis (UICC I–III) but observed no significant alteration of S100P expression in patients with or without metachronous metastasis (*P* = 0.16; *N* = 60; Fig. 5a, left). As CRC patients with CRC tumours of the UICC Stages II–III have a higher risk of distant recurrence [27], we specifically analysed this subgroup of tumour samples and indeed found a significant increase of S100P expression in tumours of patients with metachronous metastasis (*P* < 0.05; *N* = 43; Fig. 5b, left).

When we analysed the risk of the patients for metachronous metastases within 5 years after diagnosis, we observed a tendency of lower metastasis risk when S100P is low expressed in the tumours (ROC-based cut-off 3.235; Fig. 5a, centre). Again, the prognostic value of S100P becomes significant for the subgroup of patients with UICC Stages II–III (*P* = 0.03; HR = 0.37, 95% CI = 0.13–0.90; Fig. 5b, centre). In this subgroup, the risk of a patient to develop metachronous metastases is almost 70% increased when the tumour expresses high S100P levels, emphasising the value of S100P as a prognostic biomarker. Average metastasis-free survival of patients drops from 48.7 months (±3.5; 95% CI = 41.8–55.6) to 28.0 months (±4.1; 95% CI =  19.9–36.0) when S100P is highly expressed in the primary tumour. By combining the expression of S100P and MACC1 [14] for patient prognosis, we found the MACC1 expression level as a stronger indicator for increased risk of metachronous metastasis, but could also confirm a MACC1-independent prognostic value of S100P (Fig. 5a, right; MFS UICC I–III, both low 55.1 months ± 2.3; MACC1 low, S100P high 49.4 months ± 4.2), which was increased in patients with advanced localised tumours (Fig. 5b, right; MFS UICC II–III, both low 53.5 months ± 3.6; MACC1 low, S100P high 42.4 months ± 6.3). Thus, S100P can be used as a prognostic biomarker for metachronous metastasis of CRC tumour Stages II and III, and although correlated with the expression of MACC1, their combination further refines the determination of the metastatic risk of CRC patients.

## Discussion

In this study, we identified the metastasis-related gene S100P as a novel transcriptional target of MACC1. We observed MACC1-dependent S100P expression in three human CRC cell lines and confirmed the presence of MACC1 at the S100P promoter. Previous publications already report the transcriptional regulation of cancer- and metastasis-related genes by MACC1 in CRC, like the hepatocyte growth factor receptor c-MET [5, 6], the adhesion factor SPON2 [8] and the stem-cell transcription factor NANOG [7]. More putative target genes of MACC1 are identified by in silico analysis, but will need further validation [28]. We observed the binding of MACC1 to the region of −296 bp to +65 bp of the human S100P promoter, where key elements for transcription factors STAT/CREB, SMAD and SP1 are located [20]. The SP1 site in the S100P promoter is critical for the thioredoxin (TXN)-mediated S100P expression and TXN itself has been shown to interact with the S100P promoter [29]. The observed presence of MACC1 on the SP1 binding site in the promoter of the c-MET gene, which contributes to the expression of c-MET [5], strengthens the model of MACC1-driven expression of S100P. Interestingly, we identified TXN as an interaction partner of MACC1 after a pulldown in SW620 cells [30], suggesting protein complexes on the S100P promoter containing TXN and MACC1 in addition to the site-specific transcription factors.

Transcriptional regulation of S100P also occurs by prostaglandin E_2_/prostaglandin E receptor 4 signalling, where prostaglandin E_2_ induced the binding of CREB to the S100P promoter region [31]. Tothova et al. demonstrated that the glucocorticoid dexamethasone is able to induce S100P expression by binding to glucocorticoid responsive elements in the S100P promoter [32], unravelling crosstalk between the glucocorticoid receptor and the MAPK pathway. In the absence of the glucocorticoid receptor, the MAPK pathway is activated and promotes transcription of S100P via AP-1. As MACC1 overexpression indirectly leads to higher MAPK signalling by inducing c-Met expression [6, 33] this axis can also contribute to increased S100P transcription.

S100P expression is linked to cancer cell proliferation and motility and contributes to tumour progression in several solid cancers, including CRC [34–39]. Overexpression of S100P induced target genes of the NF-κB signalling, like BCL2 [40, 41] and CCND1 [42–44], potentially triggered by the interaction of S100P and RAGE [10]. The regulation of CCND1 expression by S100P has also been reported to act via nuclear localisation of β-catenin [45]. As the cell line used is mutated in the APC protein, β-catenin is accumulating in the cytoplasm [46] and a S100P-mediated nuclear translocation would sharply increase target gene expression.

Our results are also in line with the reported contribution of S100P to tumour progression and metastasis, as its ectopic expression in SW480 cells induces anchorage-dependent and -independent proliferation, cell migration and invasion. In turn, the RNAi-mediated reduction of S100P in SW620 cells decreased all the before mentioned cellular processes. Besides its impact on intracellular processes contributing to increased metastatic potential, S100P is also secreted into the intercellular fluid and can interact with several binding partners. The levels of secreted S100P in the constructed cell lines are in the range with reported low (0.1–0.2 ng/ml) or high (>0.5 ng/ml) S100P secretion [47].

The binding of S100P to the receptor of advanced glycation end products (RAGE) can trigger intracellular signalling cascades that eventually lead to increased survival of cancer cells, as well as to higher cell proliferation and migration [48, 49]. The binding of S100P to the V-domain of RAGE is rather strong, with a dissociation constant of 6.8 µM [50], and concentrations of 0.1–1000 nM S100P have been used to trigger cellular responses to extracellular S100P [48–50]. Other cancer-related interaction partners of S100P are interleukin-11 and interferon-β, and their binding can modulate local inflammation processes and the host immune response [51, 52]. For lung cancer, S100P has been identified as a critical secreted factor in the modulation of cell migration and metastasis formation [53]. In our experiments, the induction of S100P expression by transient overexpression, but also by overexpression of MACC1, resulted in increased amounts of S100P protein in the medium, where it can contribute to the increased metastatic potential of these cells.

Considering that we observed an upregulation of RAGE in MACC1-overexpressing cells, we assume a stronger S100P-mediated signalling response if the cells highly express S100P. This assumption was confirmed by the increased expression of the NF-κB target genes CCND1 and BCL2 in SW480/MACC1 genes, indicating an activated RAGE/NF-κB signalling triggered by secreted S100P [44, 48]. To test the impact of S100P expression on metastasis formation in vivo, we injected S100P- overexpressing SW480/S100P cells into the spleen of SCID beige mice and evaluated tumour growth and liver metastasis. In this experiment, cells with elevated S100P expression levels showed significantly more liver metastases, compared to SW480 control cells. Since MACC1 expression in SW480 cells or in xenograft tumours is not elevated upon S100P expression, the higher metastasis formation of SW480/S100P tumours is solely a consequence of S100P overexpression in these cells. This is in line with the observation of Ding et al., who reported higher S100P expression in liver metastases in a cell line derived xenograft assays and clinical samples [54].

Several studies showed that the S100P expression is upregulated in colorectal adenomas or carcinomas compared to mucosa tissue [31, 55–57]. S100P was significantly increased in metastasising colorectal tumours compared to non-metastasising primary tumours [54]. Our analysis of tumours from previously non-metastasised CRC patients revealed that primary tumours of patients with metachronous metastasis during the disease have a higher S100P expression than tumours from patients without metastatic relapse. S100P expression could therefore serve as a prognostic marker for metastasis-free survival of CRC patients. However, the significance of S100P expression in our tumour panel was higher in locally advanced tumours (UICC Stages II–III), leaving the prognostic value of S100P in the earlier tumour stages to be evaluated in a larger cohort before its implementation in the clinical decision.

## Supplementary information

Supplementary Table

Reproducibility checklist

## Data Availability

Data presented in this study are available on reasonable request. The datasets of MACC1-dependent differential gene expression are available at the Gene Expression Omnibus repository (GSE70458).
