# Supplementary material for: Calcium-binding protein S100P is a new target gene of MACC1, drives colorectal cancer metastasis and serves as a prognostic biomarker
Source: Br J Cancer. 2022 May 21;127(4):675–85. doi: 10.1038/s41416-022-01833-3 (PMC9381557; doi:10.1038/s41416-022-01833-3)
Supplement: Supplementary file 1 — Supplementary Table [file 41416_2022_1833_MOESM1_ESM.docx]

# Supplementary Information

**Table S1:** Origins of the parental or derived CRC cell lines used in this report.

|  | | **Expression** | | **Mutation Status** | | | | | |
| --- | --- | --- | --- | --- | --- | --- | --- | --- | --- |
| **Cell line (ATCC)** | **Source** | **MACC1** | **S100P** | **KRAS** | **BRAF** | **p53** | **APC** | **β-**  **catenin** | **MSI/**  **MSS** |
|  | *male; colon; primary* |  |  |  |  |  |  |  |  |
| *SW480 (CCL-228)* | *adenocarcinoma; Dukes B;* | +/- | + | G12V | wt | mut | mut/wt | wt | MSS |
|  | *isogenic to SW620* |  |  |  |  |  |  |  |  |
| SW480/vector | Stein et al., 2009 | +/- | + | G12V | wt | mut | mut/wt | wt | MSS |
| SW480/MACC1 | Stein et al., 2009 | ++++ | +++ | G12V | wt | mut | mut/wt | wt | MSS |
| SW480/S100P | this manuscript | +/- | ++++ | G12V | wt | mut | mut/wt | wt | MSS |
| SW480/luc/vector | this manuscript | +/- | + | G12V | wt | mut | mut/wt | wt | MSS |
| SW480/luc/S100P | this manuscript | +/- | ++++ | G12V | wt | mut | mut/wt | wt | MSS |
|  | *male; colon; lymph node* |  |  |  |  |  |  |  |  |
| *SW620 (CCL-227)* | *metastasis; Dukes C;* | ++++ | ++++ | G12V | wt | mut | mut/wt | wt | MSS |
|  | *isogenic to SW480* |  |  |  |  |  |  |  |  |
| SW620/shCtrl | Pichorner et al., 2012 | ++++ | ++++ | G12V | wt | mut | mut/wt | wt | MSS |
| SW620/shMACC1 | Pichorner et al., 2012 | + | ++ | G12V | wt | mut | mut/wt | wt | MSS |
| SW620/shS100P | this manuscript | ++++ | + | G12V | wt | mut | mut/wt | wt | MSS |
| *HT29 (HTB-38)* | *female; colon; primary adenocarcinoma; Dukes B* | ++ | ++ | wt | V600E | mut | mut | wt | MSS |
| HT29/GFP | B. Kortüm (MDC Berlin) | ++ | ++ | wt | V600E | mut | mut | wt | MSS |
| HT29/MACC1-GFP | B. Kortüm (MDC Berlin) | ++++ | ++++ | wt | V600E | mut | mut | wt | MSS |

luc – firefly luciferase; gene expression levels are indicated as +/- for very low to ++++ for very high; wt – wild type; mut – mutated; MSS – microsatellite stable

**Table S2:** S100P-specific ELISA, standard absorptions and calibration curve


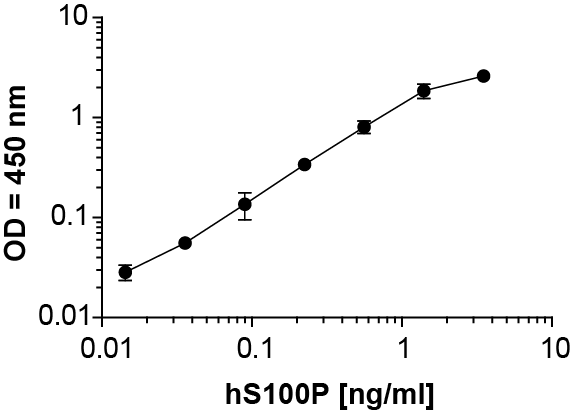


| **S100P** | **OD = 450 nm** | |
| --- | --- | --- |
| ng/ml | Average | SD |
| 3.500 | 2.605 | 0.028 |
| 1.400 | 1.857 | 0.305 |
| 0.560 | 0.808 | 0.115 |
| 0.224 | 0.340 | 0.023 |
| 0.090 | 0.136 | 0.041 |
| 0.036 | 0.056 | 0.005 |
| 0.014 | 0.029 | 0.005 |

**Table S3:** S100P-specific protein concentration in cell culture media.

| **S100P in growth medium [ng/ml]**  **Experiment 1 Experiment 2 Experiment 3 Experiment 4** | | | | | | | **cell lines** |
| --- | --- | --- | --- | --- | --- | --- | --- |
| 0.203 | 0.348 | 0.307 | 0.127 | 0.173 | 0.325 | 0.252 | SW480/MACC1 |
| 0.472 | 0.460 | 0.413 | 0.448 | 0.437 | 0.526 | 0.450 | SW480/S100P |
| 0.060 | 0.055 | 0.099 | 0.068 | 0.074 | 0.060 | 0.075 | SW480/ctrl |
| 0.186 | 0.217 | 0.340 | 0.208 | 0.247 | 0.282 | 0.284 | SW620/shMACC1 |
| 0.193 | 0.183 | 0.215 | 0.167 | 0.340 | 0.188 | 0.204 | SW620/shS100P |
| 0.846 | 1.001 | 0.849 | 1.005 | 1.032 | 1.196 | 0.796 | SW620/shctrl |
| 0.386 | 0.348 | 0.273 | 0.435 | 0.414 | 0.343 | 0.366 | HT29/GFP |
| 2.721 | 1.018 | 0.738 | 0.833 | 0.960 | 0.826 | 0.409 | HT29/MACC1-GFP |

# Supplementary References

Stein, U. *et al.* MACC1, a newly identified key regulator of HGF-MET signaling, predicts colon cancer metastasis. *Nat. Med.* **15**, 59–67 (2009).

Pichorner, A. *et al.* In vivo imaging of colorectal cancer growth and metastasis by targeting MACC1 with shRNA in xenografted mice. Clin. Exp. Metastasis **29**, 573-83 (2012).
